# Ecophysiological traits differentially modulate secondary metabolite accumulation and antioxidant properties of tea plant [*Camellia sinensis* (L.) O. Kuntze]

**DOI:** 10.1038/s41598-021-82454-3

**Published:** 2021-02-02

**Authors:** Anjan Hazra, Shrutakirti Saha, Nirjhar Dasgupta, Rakesh Kumar, Chandan Sengupta, Sauren Das

**Affiliations:** 1 Agricultural and Ecological Research Unit, Indian Statistical Institute, 203, B. T. Road, Kolkata, 700108 India; 2 Department of Life Sciences, Guru Nanak Institute of Pharmaceutical Science and Technology, Kolkata, 700114 India; 3 Darjeeling Tea Research and Development Center, Kurseong, West Bengal 734203 India; 4 Department of Botany, University of Kalyani, Nadia, 741235 India

**Keywords:** Natural variation in plants, Plant ecology, Secondary metabolism

## Abstract

Owing to the diverse growing habitats, ecophysiology might have a regulatory impact on characteristic chemical components of tea plant. This study aimed to explore natural variations in the ecophysiological traits within seasons and the corresponding multifaceted biochemical responses given by the gene pool of 22 tea cultivars. Leaf temperature and intercellular carbon concentration (Ci), which varies as a function of transpiration and net photosynthesis respectively, have significant impact on the biochemical traits of the leaf. Occurrence of H_2_O_2_, in leaves, was associated to Ci that in turn influenced the lipid peroxidation. With the increment of Ci, total phenolics, epicatechin gallate (ECG), reducing power, and radical scavenging activity is lowered but total catechin and non-gallylated catechin derivatives (e.g. epicatechin or EC, epigallocatechin or EGC) are elevated. Leaf temperature is concomitantly associated (*p* ≤ 0.01) with phenolics, flavonoids, proanthocyanidin, tannin content, reducing power, iron chelation and free radical scavenging activities. Increased phenolic concentration in leaf cells, conceivably inhibit photosynthesis and moreover, gallic acid, thereafter conjugated to catechin derivatives. This study shed light on the fundamental information regarding ecophysiological impact on the quality determining biochemical characteristics of tea, which on further validation, might ascertain the genotype selection paradigm toward climate smart cultivation.

## Introduction

Tea, the globally most consumed non-alcoholic beverage, is the infusion prepared from processed apical buds and tender leaves of the woody perennial evergreen tea plant [*Camellia sinensis* (L.) O. Kuntze]. The plant is known to grow under diverse environmental conditions, from tropical warm and humid to temperate cold regions. Besides, tea plant also serve as an efficient and sustainable carbon sink for their dense green coverage along mountain slopes and valleys^1^. Hence, being a long lifespan (over 150 years) tree plant and evolved to thrive in diverse ecological condition, tea plant represent an ideal model for harbouring adaptation to climate change as well. Earlier report suggested that elevated CO_2_ level, in experimental condition, exerted significant impact on tea quality^2^. Li et al.^3^ reported that rising CO_2_, improves the primary metabolism of tea plant, as well as promotes secondary metabolites towards production of the superior quality green tea.

Enormous health benefits and refreshing traits of tea are attributed to the incidences of some typical secondary metabolites. Principally, three major classes including polyphenols, amino acids and alkaloids collectively account for 35–50% of its dry weight which offer some unique health benefit trait on consumption^4,5^. These particular biochemical traits are sensitive to environmental variations and also support the plant to confer certain stress tolerance ability^6,7^. Thereby the quality parameters of tea should essentially be modulated by the ecophysiological responses of the plant. It is assumed that—in the scenario of global climate change people would having tea varieties, which in the course of adaptation might be more tastier and healthier than what at present^8^.

Photosynthetic aptitude can be considered as a key driver of plant secondary metabolism for mediating ecological interactions through chemical defence^9^ which in turn, would certainly be influenced by genotype of the plant. Complex relationship between photosynthesis and the occurrences of secondary metabolites has already been well established in plant^9,10^. Genotypic variation is one of the key factors by which the plant can differentially respond to a particular environmental stimuli^11^. In tea, challenges remained viable to explore the genetic diversity underlying varying physiological response to environment^12,13^. This study aimed to explore natural variations in the ecophysiological traits within seasons and the corresponding multifaceted biochemical responses given by the gene pool of 22 tea cultivars. Thus, the common trend line of primary and secondary metabolism within the species *Camellia sinensis* would be drawn in a particular agro-ecological condition. Conferring to present study, accurate information about the dynamicity and interrelationships of such characteristics across growing seasons or genotypes, would substantiate the cultivar selection process for subsequent plantation program or harvesting time calibration towards a sustainable tea industry.

## Materials and methods

### Study site and plant materials

A total of twenty two cultivars of tea were selected for this study, which are being maintained at Darjeeling Tea Research and Development Centre (DTR & DC), Kurseong, West Bengal, India for research and breeding purposes. These tea cultivars were propagated together, using randomized complete block design at the experimental field of DTR & DC and grown under the uniform agro-ecological conditions. Plants with ten years of maturities were selected for the present study on the basis of their contrasting agronomic traits reported earlier^14^ (Table 1). The study site is located between 26.88ʺ 21° N, 88.27ʺ 89° E, a part of lower eastern Himalaya with an elevation of 1530 m above sea level. The topography of the field comprises moderate slopes (25–45%). The annual average rainfall of this area is 3235 mm and the temperature varies from 8 to 20 °C.

**Table 1:** Selected cultivars and their main agro-morphological properties.

Cultivars	Abbreviated as	Group	Yield	Flavour
Amberi Valai-2	AV-2	China hybrid	1500–2500 kg/ha	+++
Athrey (UPASI-9)	B/6/61	Assam variety	NA	NA
Badamtam-15/263	B/15/263	China hybrid	1500–2500 kg/ha	++
Balasun-7/1A/76	BS/1A/76	China hybrid	1500–2500 kg/ha	++
Bannockburn-157	B-157	Cambod type	< 1500 kg/ha	++
Bannockburn-668	B-668	Assam hybrid	< 1500 kg/ha	+++
Bannockburn-777	B-777	China hybrid	< 1500 kg/ha	+
Happy Valley-39	HV-39	China hybrid	2500–3000 kg/ha	+
Kopati-1/1	K-1/1	Assam hybrid	< 1500 kg/ha	+++
Makaibari-6	MB-6	Not available	1500–2500 kg/ha	++
Nandadevi (TS-378)	TS-378	China hybrid	< 1500 kg/ha	++
Pandian (UPASI-10)	B/6/62	China variety	NA	NA
Phoobsering-312	P-312	China hybrid	1500–2500 kg/ha	++
Rungli Rungliot-17/144	RR/17/144	China hybrid	2500–3000 kg/ha	++
Sikkim-1	S-1	China hybrid	1500–2500 kg/ha	++
Springfield (UPASI-15)	SP/4/5	China variety	NA	NA
Sundaram (UPASI-3)	B/5/63	Assam hybrid	2500–3000 kg/ha	+
Teesta Valley-1	TtV-1	China hybrid	1500–2500 kg/ha	+
Tukdah-135	T-135	Assam hybrid	< 1500 kg/ha	+
Tukdah-253	T-253	Assam type	1500–2500 kg/ha	++
Tukdah-383	T-383	China hybrid	1500–2500 kg/ha	+++
Tukdah-78	T-78	China hybrid	2500–3000 kg/ha	++

+, ++ and +++ are denoted as average, good, and excellent flavour respectively.

*NA *information not available.

### Measurement of stomatal index

The stomatal indices of the selected cultivars were determined from epidermal peelings of the leaves (ten from each genotype) using a compound microscope. Fresh leaves were collected from the field and fixed in 70% ethanol. Number of stomata and epidermal cell count per unit area from apical, middle and basal regions (ten readings from each part of leaves) were recorded under 10 × and 20 × objectives of the microscope. Photomicrographs were taken with the help of a trinocular phase contrast microscope (Axiolab Zeiss Inc.) equipped with a Motic 3.0 digital camera.

### Photosynthetic parameters

The ecophysiological measurements [Net photosynthetic rate (Pn), transpiration (E), stomatal conductance (gs), water use efficiency (WUE), vapour pressure deficit (VPD), relative humidity (RH), leaf temperature (LT), and intercellular CO_2_ concentration (Ci)] were determined using a portable infrared gas analyzer (Li 6200, Licor Inc., Nebraska, USA) equipped with a 390 cm^3^ chamber. Five bushes per genotype with dark-green, sunlight exposed maintenance leaves were randomly chosen for these data collection during three commercial plucking seasons (spring, rain and autumn). Leaf surfaces were cleaned and dried using tissue paper prior to enclose the leaf in cuvette. Before each measurement, the instrument was warmed for 15 min and calibrated to zero. Optimal conditions for the leaf cuvette were set to 30 °C temperature and 60% relative humidity. After achieving CO_2_ concentration of the chamber near ambient (i.e., 400 μmol), the log program was initiated and three such observations were recorded accordingly. The measurements of gas exchange were carried out between 10:00 and 11:30 AM. Accomplished data in the field was retrieved using ‘HyperTerminal’ windows program.

### Determination of biochemical traits

Representative samples were collected from the experimental field during three commercial plucking seasons (spring, rain and autumn) in the year 2017. Fresh and healthy shoots (one apical bud and two adjoining leaves) were hand-plucked from each bushes of the 22 cultivars from which the photosynthetic data were collected. Samples were immediately stored in frozen condition and subsequently transferred to the laboratory for long term storage and downstream experimental works. A pool of harvested shoots from the five bushes of each cultivar was taken into account for biochemical assessment. For each analysis, three replicates were considered. Samples were extracted following the methodology reported earlier^15^ and stored at − 20 °C for further analyses. Quantification of various secondary metabolites, assessment of total antioxidant power, ROS/RNS scavenging ability, metal chelating ability, H_2_O_2_ contents, lipid peroxidation activity and reducing power of the studied samples were determined using a UV–Vis spectrophotometer (Helios λ, Thermo Scientific Corporation) based on the standard reference protocols (see Supplementary Table 1 for reference of protocols adopted)^5^. For determination of catechin derivatives, high-performance liquid chromatography was carried out in a Waters Alliance MDLC Separations module and dual absorbance detector (Waters e2695-2487) equipped with a C18 reverse-phase silica-based column (X-Tera) with 4.6 × 250 mm dimension at 35 °C. The double gradient elution system was followed using standardized methodology^16^. Standard reference materials of various catechin derivatives were used (Sigma-Aldrich, stored at − 20 °C and diluted from the stock for using as working solution). Catechins and catechin derivatives and caffeine peaks were identified and enumerated through comparing the retention times and peak areas obtained from standard reference materials using empower pro software (Waters Corporation).

### Statistical analyses

All the experimental data were acquired following the principle of replication^17^. At least five individuals per genotype were considered for each assessment and biochemical measurements were repeated thrice. Mean values from observations of parameters for each genotype in every plucking season was considered as representative data point. Hierarchal clustering and heatmap generation was performed following Metsalu and Vilo^18^. Raw data points were normalised with unit variance scaling and correlation distances were measured for clustering. Pearson’s bivariate correlations coefficients were estimated to depict the possible association between the ecophysiological and biochemical traits. Regression plot, distribution histogram and correlation coefficient values are demonstrated employing ‘corrplot’ and ‘Performance Analytics’ packages implemented with the R statistical program 3.6.2^19^.

## Results

**Figure 1:**
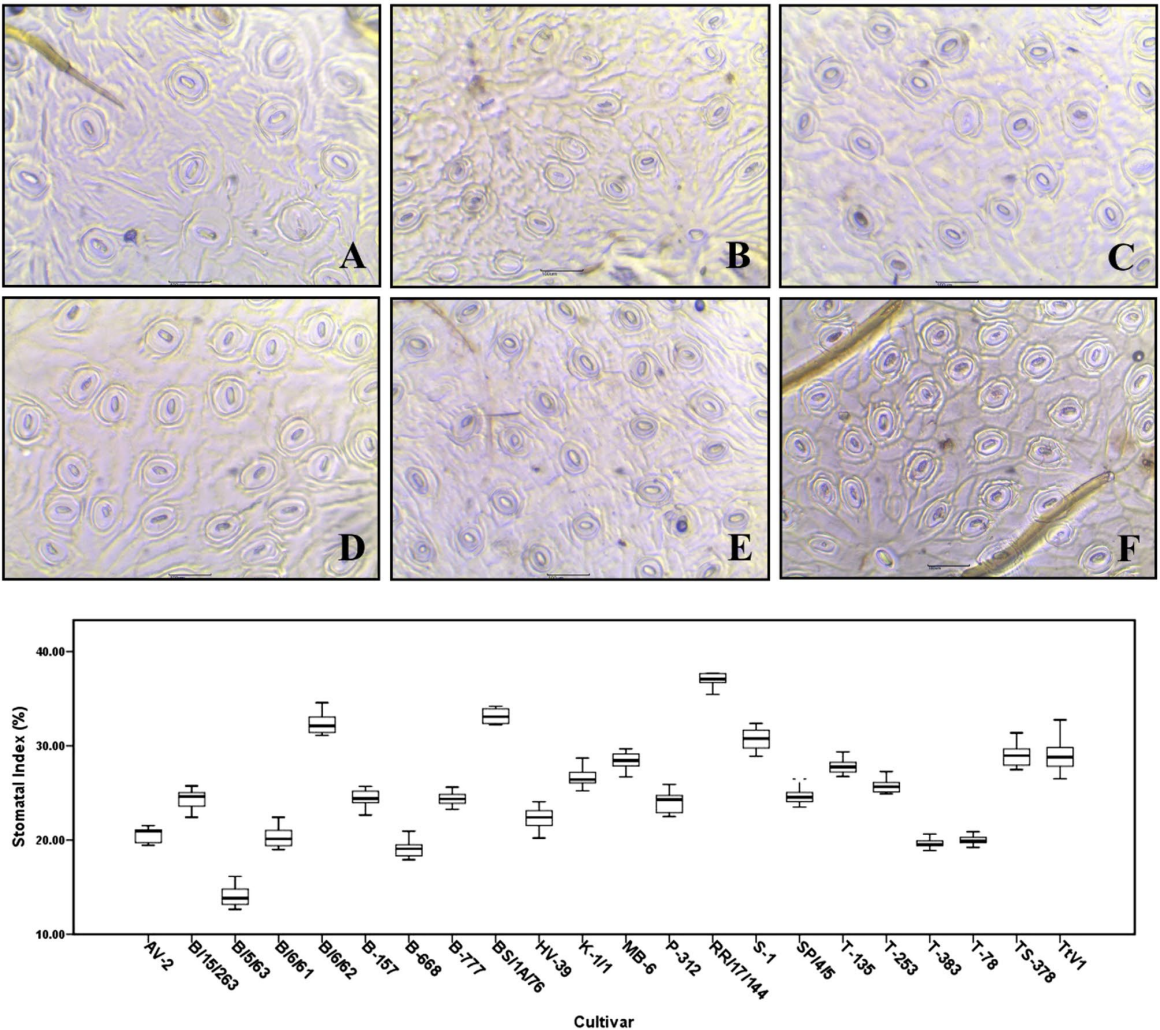
Measurements of stomatal indices; ventral epidermal surfaces of representative cultivars, (**A**) B/5/63, (**B**) AV2, (**C**) B668, (**D**) K1/1, (**E**) P312, and (**F**) RR/17/144; box plot showing mean stomatal indices for studied 22 genotypes.

**Figure 2:**
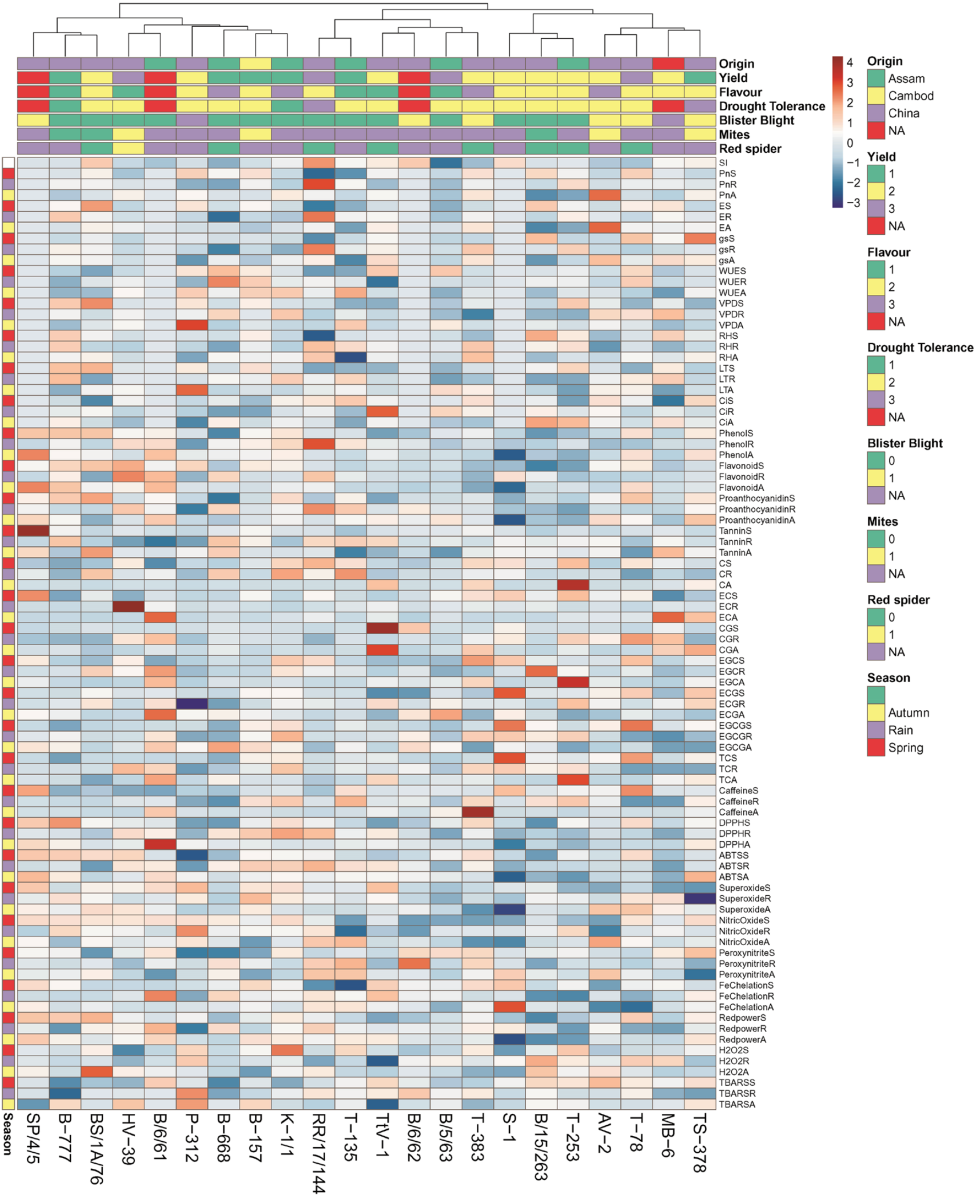
Hierarchical clustering showing incidences of the studied ecophysiological and biochemical variables (22 genotypes × 3 seasons). Rows are centered with unit variance scaling. Imputation is used for missing value estimation. Columns are clustered using correlation distance and Ward linkage. Agronomic characters of the genotypes^14^ are denoted as—Yield: (1) < 1500 kg/ha, (2) 1500–2500 kg/ha, (3) 2500–3000 kg/ha; Flavour: (1) Average, (2) Good, (3) Excellent; drought tolerance: (1) Fair, (2) Good, (3) Very good; Blister blight, mites and red spider: (1) Resistant, (0) susceptible; *NA* data not available. Heatmap generation and hierarchical clustering have been carried out using ClustVis^18^.

Among the studied 22 cultivars, RR/17/144, B/6/62, and BS/1A/76 possess higher stomatal indices than others and it was lowest in the triploid cultivar B/5/63 (Fig. 1). Net photosynthesis rate, however, in RR/17/144 is lowest in spring season and maximum in rain season. In few other cultivars (like P-312, B/15/263, and T-78) the trend is opposite. In AV-2, the maximum photosynthesis rate occurs during autumn season. It is evident that the high yielding cultivars are mostly with lower stomatal indices, except RR/17/144. Lower stomatal index, in turn, is generally associated to reduce rate of photosynthesis and transpiration. Though, RR/17/144 with high stomatal index, maintains lower rate of transpiration (as well as net photosynthesis rate) at summer and AV-2, with low stomatal index, shows remarkable high photosynthesis thereby transpiration during autumn season. Lowest relative humidity (RH) observed in RR/17/144 (during spring) and T-135 (during autumn). BS/1A/76 and RR/17/144 shows maximum rate of transpiration in spring and rainy seasons respectively. In autumn season, vapour pressure deficit (VPD) and leaf temperature (LT) reaches its peak in P-312. In most of the seasons, B/6/61, B-668, and B-157 sustains high to moderate water use efficiency (WUE) consistently (Fig. 2).

An overview of the existing diversity in ecophysiological parameters and relative abundances of secondary metabolites is presented in Fig. 2. Secondary metabolite contents and corresponding in vitro antioxidant activities in all studied cultivars were observed to attain their peak value during the rainy season. During spring flush, total phenols content is most abundant in BS/1A/76 and, occurrence of total phenol is highest during rainy season in RR-17/44 (Fig. 2). Minimum occurrence of total phenol in B-668 during spring, but in rainy season the least amount was recorded in AV-2. In autumn, the highest and lowest total phenol noted in SP-4/5 and S-1 respectively. A remarkably high amount of total flavonoid occurred in the mid-season (rainy) in all cultivars and it ranged between the amount in HV-39 and P-312. In autumn flush, all cultivars showing declining trends than those amounts of the rainy season, except in P-312, where the value decreases nominally. In the spring season, the assayed proanthocyanidin is ranged within the amount in BS/1A/76 and TtV-1. In rainy season, the highest amount occurred in RR-17/144 and lowest occurred in P-312 and in last flush (autumn flush) highest value obtained in B-6/61 and lowest in S-1. During springtime, the differences of tannin occurrence among all the cultivars are marginal. Plant sampling in rainy season showed the predominant amount of tannin in BS-1A/76, and lowest in T-78 cultivar.

**Figure 3:**
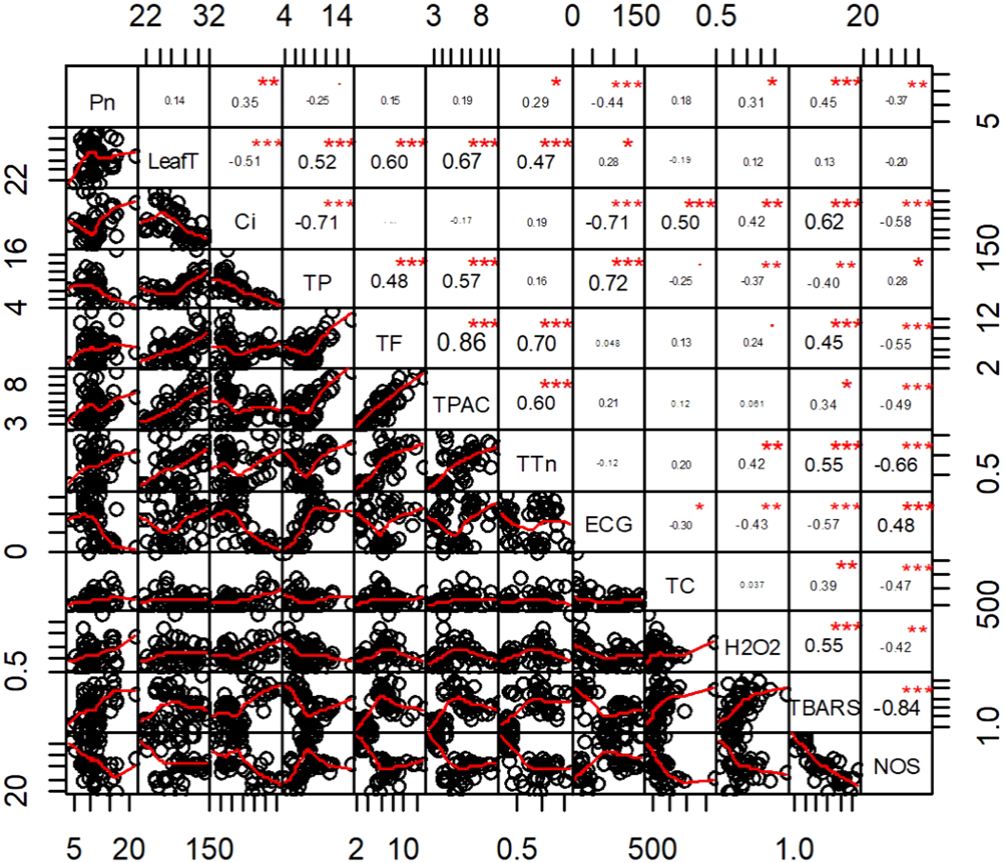
Correlation coefficients and linear regression among the ecophysiological and biochemical variables. Bottom of the diagonal shows the bivariate scatter plots with a fitted line. Top of the diagonal indicates the Pearson’s bivariate correlation value with the significance level as stars (*p* values ≤ 0.001, ≤ 0.01, and ≤ 0.05 are denoted as ‘***’, ‘**’, and ‘*’ respectively). Correlation plot was prepared with the help of ‘corrplot’ and ‘Performance Analytics’ packages implemented with the R package v3.6.2.

Free radical scavenging or antioxidant activities such as for ABTS and DPPH ions, the values are mostly in concomitant to the gradient of secondary metabolites contents of tissues. Nitric oxide and Peroxynitrite scavenging ability of the tea samples, however, are negatively related to the flavonoid, proanthocyanidin and tannin concentration (Fig. 2). Also, nitric oxide and Peroxynitrite radical scavenging activities consistently showed a strong reverse correlation with the malondealdehyde content, the end product of lipid peroxidation (Fig. 3, Supplementary Table 2). Among the catechin derivatives epigallocatechin gallate (EGCG) is the most prevalent followed by ECG. Owing to the phenolic compounds, EGCG and ECG are positively correlated with studied secondary metabolites and radical scavenging activities, in most of the cultivars (except S-1, B-777). Nevertheless, the concentration of minor catechin derivatives (i.e. C, EC and EGC) is in contrary with the total phenolics contents, DPPH, nitric oxide, Peroxynitrite scavenging activities and the ECG, EGCG amount itself within the tissue.

When comparing ecophysiological traits of the leaves with occurrence of secondary metabolites and antioxidant activities therein, it shows remarkable associations with sound statistical validity (Fig. 3, Supplementary Table 2). The comparison between every studied parameters revealed, leaf temperature (LT) and intercellular carbon concentration (Ci) have significant impact on the antioxidant related biochemical traits of the leaf. It showed the significant reverse correlation between Ci and phenolic contents and on the other hand, positive correlation of phenolics with LT. Total catechin, though, elevated with Ci, some of its derivatives (especially ungallylated catechins) negatively regulated with increasing Ci. Amounts of H_2_O_2_, overall, was associated to Ci and influenced the lipid peroxidation activity. With the increment of Ci, total phenolics, ECG, reducing power, DPPH and NO radical scavenging activity is lowered and besides lipid peroxidation activity also elevated. Leaf temperature is concurrently associated with phenolics, flavonoids, proanthocyanidin, tannin content, reducing power, iron chelation and ABTS, DPPH scavenging activities. Moreover, the ECG content and Peroxynitrite, nitric oxide scavenging activities are to some extent vary as a function of rate of gas exchange and transpiration.

## Discussion

This study examined the natural diversity and interrelationships among the ecophysiological traits belonged to the 22 cultivars and the secondary metabolite composition they express simultaneously. Stomatal indices, here, showed near linear relationship with photosynthesis and transpiration during rainy season, while moisture content is adequate in the atmosphere. In spring season, however, genotypic variations responded differentially to the CO_2_ assimilation process. Assam type cultivars (e.g. B668, K-1/1, B/6/61, T253, etc.), which are considered to be the adapted to the tropical warm climate, are showing lower stomatal density. The drought tolerant cultivars like RR/17/144 also check their transpiration rate to a certain level under moisture deficit conditions during summer. The moderately drought susceptible cultivar T-78^14^ with lower stomatal index, contrarily, shows high photosynthetic behaviour during spring time and the trend is reversed during monsoon. In T-78, high CO_2_ assimilation rate leading to increased phenol, flavonoid and catechin content in spring season, all of which just down-regulated in a similar trend during monsoon. This observation is consistent with the previous experiment where CO_2_ enrichment remarkably increased the level of polyphenol contents including catechins^3^. According to this carbon-nutrient hypothesis, as CO_2_ enrichment increased the carbon to nitrogen ratio in leaves, thus a major amount of carbon could be allocated to production of C-based secondary metabolites such as catechins^20^. The overall trend from all genotype observations in present analyses exhibited that, total catechin accumulation in tea leaves increases alongside the increasing rate of photosynthesis. Afterwards this lead to elevation of total phenolics and radical scavenging activity. Among the catechin derivatives, catechin monomer and epicatechins (EC, EGC) are elevated together with intercellular CO_2_ concentration, interestingly, their gallylated members (ECG, EGCG) are not. The present observation revealed that H_2_O_2_ act as a key signalling molecule which is significantly associated (*p* ≤ 0.01) to intercellular CO_2_ concentration and concomitantly triggered lipid peroxidation of leaf. It is suggested that increased level of H_2_O_2_ and MDA might be involved in the up-regulation of the secondary metabolites production^21^. According to this study, abundance of H_2_O_2_ and MDA contents observed in the samples with lower phenolics and radical scavenging activity but with higher tannin and catechin concentrations.

The concentration of catechin and methylxanthine secondary metabolites, which is considered as primarily tea quality determining factor, is adversely lowered during monsoon in comparison to spring season, whereas, total phenolic concentration and antioxidant activity increased at the same time^22^. In this study, second flush samples that harvested during rainy season, revealed highest amount of phenolic contents, proanthocyanidin and catechin among all seasons. This seasonal prevalence could be influenced by an array of biotic and abiotic factors. Ercisli et al.^23^ reported similar kind of findings where phenolic contents and antioxidant activity of tea leaves elevated during second flush than first and third ones. They concluded this was due to atmospheric temperature, day length and sunlight effect where shade effect and moisture content had an important role in phenolic compound synthesis in the plant. Erturk et al.^24^ suggested that the level of total phenols in tea leaves augmented throughout warmer months from July to September. Elevated levels of UV irradiance also considered to be responsible for the up-regulation of different flavonoids which contribute to the flavour and antioxidant aptitude of Darjeeling tea^25^. In another report, tea quality determining compounds were said to occur best in the second flush due to synthesis and accumulation of excess amount of secondary metabolites in leaves and buds during that time^26^. This abundance on the extent of secondary metabolites was mostly induced by the infestation of jassids and thrips, which imposed mechanical stress on host plants^26^. The attack of Jassids and thrips commences during the onset of monsoon in Darjeeling hills^27,28^, and this also might be an important reason behind the elevated level of secondary metabolite in tea shoots sampled during the rainy season.

Gulati et al.^29^ suggested that the biochemical representation of tea plants with various proportion of catechin and its components is useful in the quality-tea clone production process. Non-esterified catechins are transformed to its esterified derivatives in course of flavan-3-ol biosynthesis^30^. According to the evolutionary process of flavan-3-ol biosynthesis of tea leaves, the constitution of non-esterified types of catechins i.e. EC and C generally occur considerably high in primitive type of tea plants^31^. Li et al.^31^ postulated that the greater the amount of EGCG and EGC with lower proportion of EC and C indicates the more recent origin of the tea tree lines. In present study, among the catechin and catechin derivatives, EGCG content was highest in amount followed by ECG, which is in conformity with the earlier studies^32–34^. Seasonal variation revealed the remarkable variation of the above. Yao et al.^35^ opined that EGC level in tea become higher in the cooler months, and EGCG, ECG become elevated during warmer months. In this study, EGC content in all the cultivars elevated from spring to autumn, ECG content of 16 cultivars out of 22 showed highest in second flush. EGCG contents, however, revealed wide diversity along with seasonal variation. Similar occurrence was also observed by Fang et al.^36^ where EGCG content did not vary across the seasons and they concluded that responses of all cultivars are not same in respect of EGCG content due to the interaction of climatic conditions and cultivar origin (genotype) might influence on observed data across the seasonal changes. Nonetheless, occurrence of phenolics and EGCG in a complementary manner is consistent with the previous observation^37^. Moreover, Assam type tea cultivars, predominantly, in this study possessed higher amount of C and EC which can be attributed to their comparatively primitive origin. Notably, phylogenetic status of the studied cultivars were already established in a recent study that grouped the genotypes into Assam and China type clade^38^. The information of dynamicity in their catechin fraction, obtained in present study, would also assist the genotype selection process toward development of the superior tea cultivars with enriched health benefit traits. Accordingly, cultivars of putatively Assam type origin such as K-1/1, B/6/61, B-668 etc. which also portrayed with potential health benefit and ecophysiological traits, should be considered in this regard.

Concerning secondary metabolism in tea, the role of photosynthetic parameters could be indicated. Earlier report stated that phenolic acids could have some inhibitory effect on photosynthesis^39^. The present findings revealed that with elevated intercellular CO_2_, net photosynthesis rate is increased that is further positively associated to catechin synthesis. Afterwards, the excess excitation energy in the photosynthetic electron transport chain is transferred to oxygen and lead to the formation of hydrogen peroxide^9^. This H_2_O_2_, being a reactive oxygen species, caused lipid peroxidation in the leaves which in turn, triggered the phenolics and flavonoids synthesis pathway. Later on, increased phenolic concentration in leaf cells, conceivably inhibit photosynthesis as postulated earlier^39^. Gallic acid, one of the phenolic acid, meanwhile conjugated to C, EC, EGC and give rise to CG, ECG and EGCG respectively. That is why gallylation of tea catechins have been reported to be influenced by cultivar and seasons too^40^. This proposed pathway, however, needs to be validated by examining the responses by tea plants grown along CO_2_ regime along with differential expression analyses of the related primary and secondary metabolism pathway genes. In summary, observations of this study highlight the information regarding ecophysiological impact on the quality determining biochemical characteristics of tea, which on further validation, might ascertain the climate induced system biology aspects.

## Supplementary Information

Supplementary Information.
