# Supplementary material for: Ecophysiological traits differentially modulate secondary metabolite accumulation and antioxidant properties of tea plant [Camellia sinensis (L.) O. Kuntze]
Source: Sci Rep. 2021 Feb 2;11:2795. doi: 10.1038/s41598-021-82454-3 (PMC7854609; doi:10.1038/s41598-021-82454-3)
Supplement: Supplementary file 1 — Supplementary Information. [file 41598_2021_82454_MOESM1_ESM.doc]

**Ecophysiological traits differentially modulate secondary metabolite accumulation and antioxidant properties of tea plant [*Camellia sinensis* (L.) O. Kuntze]**

Anjan Hazra^1^, Shrutakirti Saha^1^, Nirjhar Dasgupta^2^, Rakesh Kumar^3^, Chandan Sengupta^4^, Sauren Das^1^*

^1^Agricultural and Ecological Research Unit, Indian Statistical Institute, 203, B. T. Road, Kolkata 700108, India

^2^Department of Life Sciences, Guru Nanak Institute of Pharmaceutical Science and Technology, Kolkata-700114, India

^3^Darjeeling Tea Research and Development center, Kurseong, West Bengal 734203, India

^4^Department of Botany, University of Kalyani, Nadia 742325, India

*Corresponding author: [sauren@isical.ac.in](mailto:sauren@isical.ac.in)

**Supplementary table 1: Parameters considered for antioxidant traits**

| **Parameters** | **References** |
| --- | --- |
| ***Secondary metabolites*** |  |
| Phenolics | Velioglu et al., 1998 |
| Flavonoids | Zhishen et al., 1999 |
| Proanthocyanidin | Sun et al., 1998 |
| Tannin | Dasgupta et al., 2017 |
| ***Total antioxidant ability*** |  |
| DPPH quenching | Blois, 1958 |
| ABTS quenching | Re et al., 1999 |
| ***Reducing power*** | Ozaizu, 1986 |
| ***Ferrous ion chelation activity*** | Haro-Vicente et al., 2006 |
| ***ROS/RNS scavenging*** |  |
| Superoxide radical | Fontana et al., 2001 |
| Nitric oxide radical | Garrat, 1964 |
| Peroxynitrite radical  H_2_O_2_ content | Bailly et al., 2000  Junglee et al., 2014 |

**References**

Bailly, F., Zoete, V., Vamecq, J., Catteau, J.-P., & Bernier, J.-L. (2000). Antioxidant actions of ovothiol‐derived 4‐mercaptoimidazoles: glutathione peroxidase activity and protection against peroxynitrite‐induced damage. Febs Letters, 486(1), 19-22.

Blois, M. S. (1958). Antioxidant determinations by the use of a stable free radical. Nature, 181(4617), 1199.

Dasgupta, N., Nandy, P., Sengupta, C., & Das, S. (2017). Occurrence of Secondary Metabolites and Free Radical Scavenging Ability towards Better Adaptability of Some Mangrove Species in Elevated Salinity of Indian Sundarbans. Annals of Tropical Research, 39(1), 13-38.

Fontana, M., Mosca, L., & Rosei, M. A. (2001). Interaction of enkephalins with oxyradicals. Biochemical Pharmacology, 61(10), 1253-1257.

Garratt, D. C. (1964). The quantitative analysis of Drugs. Volume 3. : Chapman and Hall ltd, Japan. .

Haro-Vicente, J., Martinez-Gracia, C., & Ros, G. (2006). Optimisation of in vitro measurement of available iron from different fortificants in citric fruit juices. Food chemistry, 98(4), 639-648.

Junglee, S., Urban, L., Sallanon, H., & Lopez-Lauri, F. (2014). Optimized assay for hydrogen peroxide determination in plant tissue using potassium iodide. *American Journal of Analytical Chemistry*, *5*(11), 730.

Oyaizu, M. (1986). Studies on products of browning reaction. The Japanese journal of nutrition and dietetics, 44(6), 307-315.

Re, R., Pellegrini, N., Proteggente, A., Pannala, A., Yang, M., & Rice-Evans, C. (1999). Antioxidant activity applying an improved ABTS radical cation decolorization assay. Free Radical Biology and Medicine, 26(9-10), 1231-1237.

Sun, B., Ricardo-da-Silva, J. M., & Spranger, I. (1998). Critical factors of vanillin assay for catechins and proanthocyanidins. Journal of agricultural and food chemistry, 46(10), 4267-4274.

Velioglu, Y., Mazza, G., Gao, L., & Oomah, B. (1998). Antioxidant activity and total phenolics in selected fruits, vegetables, and grain products. Journal of agricultural and food chemistry, 46(10), 4113-4117.

Zhishen, J., Mengcheng, T., & Jianming, W. (1999). The determination of flavonoid contents in mulberry and their scavenging effects on superoxide radicals. Food chemistry, 64(4), 555-559.

**Supplementary table 2: Pearson’s correlation coefficients among the studied traits**

|  | SI | PPFD | Pn | E | gs | WUE | VPD | RH | LTH | Ci | Phenol | Flavonoid | Proanthocyanidin | TanninJ | Redpower | DPPH | ABTS | FeChelation | NitricOxide | Peroxynitrite | Superoxide | TBARS | EGC | Caffeine | C | EC | EGCG | ECG | CG | TC |
| --- | --- | --- | --- | --- | --- | --- | --- | --- | --- | --- | --- | --- | --- | --- | --- | --- | --- | --- | --- | --- | --- | --- | --- | --- | --- | --- | --- | --- | --- | --- |
| SI | 1 | -0.005 | 0.045 | 0.181 | 0.166 | -0.191 | -0.036 | 0.065 | -0.012 | 0.019 | 0.157 | 0.064 | 0.027 | 0.077 | 0.015 | 0.119 | 0.05 | 0.125 | 0.097 | -0.018 | 0.085 | -0.023 | -0.048 | -0.185 | 0.049 | 0.087 | -0.189 | -0.009 | 0.039 | -0.028 |
| PPFD | -0.005 | 1 | .285* | .368** | -0.051 | -0.067 | .442** | 0.195 | .546** | -.334* | 0.196 | 0.055 | 0.084 | 0.022 | 0.092 | 0.115 | 0.07 | 0.187 | 0.095 | -0.004 | 0.013 | -0.118 | 0.094 | 0.041 | 0.087 | -0.228 | -0.059 | 0.134 | -0.042 | -0.054 |
| Pn | 0.045 | .285* | 1 | .792** | .733** | .422** | -0.253 | .649** | 0.136 | .349** | -0.25 | 0.146 | 0.193 | .288* | -0.219 | -0.139 | 0.165 | -0.049 | -.372** | -.391** | 0.201 | .448** | .276* | .328* | 0.142 | .285* | -0.102 | -.439** | -0.134 | 0.176 |
| E | 0.181 | .368** | .792** | 1 | .732** | -0.172 | -0.037 | .637** | .418** | 0.16 | 0.035 | .387** | .449** | .491** | 0.151 | 0.073 | .388** | -0.136 | -.403** | -.514** | .470** | .379** | 0.157 | .292* | 0.085 | .302* | -0.061 | -0.161 | -0.165 | 0.116 |
| gs | 0.166 | -0.051 | .733** | .732** | 1 | 0.086 | -.659** | .796** | -0.214 | .638** | -.387** | 0.094 | 0.087 | .306* | -.328* | -.291* | 0.061 | -0.216 | -.461** | -.320* | 0.14 | .463** | .390** | .342* | .306* | .537** | -0.232 | -.483** | -0.212 | .381** |
| WUE | -0.191 | -0.067 | .422** | -0.172 | 0.086 | 1 | -.292* | 0.127 | -.321* | 0.235 | -.393** | -0.256 | -.285* | -0.223 | -.497** | -.276* | -0.242 | 0.178 | 0.006 | 0.059 | -.312* | 0.157 | 0.19 | 0.068 | 0.097 | 0.005 | -0.032 | -.402** | 0.094 | 0.125 |
| VPD | -0.036 | .442** | -0.253 | -0.037 | -.659** | -.292* | 1 | -.508** | .818** | -.737** | .599** | .344* | .414** | 0.143 | .676** | .566** | .391** | 0.207 | 0.193 | -0.121 | .315* | -0.228 | -.340* | -0.177 | -0.268 | -.363** | 0.214 | .510** | 0.11 | -.343* |
| RH | 0.065 | 0.195 | .649** | .637** | .796** | 0.127 | -.508** | 1 | 0.045 | .566** | -.351** | 0.233 | 0.153 | .415** | -0.255 | -0.208 | 0.215 | -0.123 | -.628** | -.455** | 0.185 | .614** | .427** | .369** | .308* | .394** | -0.061 | -.539** | -0.252 | .388** |
| LTH | -0.012 | .546** | 0.136 | .418** | -0.214 | -.321* | .818** | 0.045 | 1 | -.509** | .517** | .604** | .672** | .467** | .703** | .582** | .668** | 0.15 | -0.2 | -.476** | .543** | 0.133 | -0.177 | 0.063 | -0.172 | -0.147 | 0.24 | .278* | -0.054 | -0.191 |
| Ci | 0.019 | -.334* | .349** | 0.16 | .638** | 0.235 | -.737** | .566** | -.509** | 1 | -.712** | -0.036 | -0.168 | 0.19 | -.584** | -.532** | -0.126 | -.328* | -.581** | -.301* | -0.017 | .623** | .567** | 0.181 | .424** | .493** | -0.201 | -.706** | -.290* | .504** |
| Phenol | 0.157 | 0.196 | -0.25 | 0.035 | -.387** | -.393** | .599** | -.351** | .517** | -.712** | 1 | .478** | .566** | 0.157 | .760** | .847** | .536** | .323* | .282* | 0.055 | .313* | -.400** | -.429** | -0.215 | -.302* | -.290* | .371** | .724** | 0.095 | -0.25 |
| Flavonoid | 0.064 | 0.055 | 0.146 | .387** | 0.094 | -0.256 | .344* | 0.233 | .604** | -0.036 | .478** | 1 | .859** | .702** | .663** | .595** | .861** | 0.034 | -.548** | -.716** | .709** | .452** | 0.025 | -0.032 | 0.002 | 0.2 | .282* | 0.048 | -0.132 | 0.126 |
| Proanthocyanidin | 0.027 | 0.084 | 0.193 | .449** | 0.087 | -.285* | .414** | 0.153 | .672** | -0.168 | .566** | .859** | 1 | .597** | .764** | .706** | .933** | 0.088 | -.493** | -.643** | .638** | .344* | 0.012 | 0.139 | 0.004 | 0.163 | .282* | 0.209 | -0.126 | 0.116 |
| TanninJ | 0.077 | 0.022 | .288* | .491** | .306* | -0.223 | 0.143 | .415** | .467** | 0.19 | 0.157 | .702** | .597** | 1 | .485** | .302* | .686** | 0.075 | -.661** | -.723** | .643** | .551** | 0.159 | 0.109 | 0.111 | .320* | 0.13 | -0.117 | -0.163 | 0.201 |
| Redpower | 0.015 | 0.092 | -0.219 | 0.151 | -.328* | -.497** | .676** | -0.255 | .703** | -.584** | .760** | .663** | .764** | .485** | 1 | .740** | .754** | 0.184 | -0.103 | -.383** | .600** | -0.054 | -0.26 | -0.097 | -0.178 | -0.167 | .376** | .588** | 0.086 | -0.133 |
| DPPH | 0.119 | 0.115 | -0.139 | 0.073 | -.291* | -.276* | .566** | -0.208 | .582** | -.532** | .847** | .595** | .706** | .302* | .740** | 1 | .766** | .410** | -0.02 | -0.167 | .364** | -0.086 | -.339* | -0.072 | -0.228 | -0.152 | .441** | .500** | -0.023 | -0.114 |
| ABTS | 0.05 | 0.07 | 0.165 | .388** | 0.061 | -0.242 | .391** | 0.215 | .668** | -0.126 | .536** | .861** | .933** | .686** | .754** | .766** | 1 | 0.152 | -.559** | -.686** | .666** | .426** | 0.004 | 0.122 | -0.003 | 0.144 | .383** | 0.152 | -0.135 | 0.146 |
| FeChelation | 0.125 | 0.187 | -0.049 | -0.136 | -0.216 | 0.178 | 0.207 | -0.123 | 0.15 | -.328* | .323* | 0.034 | 0.088 | 0.075 | 0.184 | .410** | 0.152 | 1 | 0.181 | 0.162 | -0.182 | -0.258 | -0.158 | -0.049 | -0.038 | -0.139 | 0.015 | 0.184 | 0.254 | -0.085 |
| NitricOxide | 0.097 | 0.095 | -.372** | -.403** | -.461** | 0.006 | 0.193 | -.628** | -0.2 | -.581** | .282* | -.548** | -.493** | -.661** | -0.103 | -0.02 | -.559** | 0.181 | 1 | .790** | -.434** | -.841** | -.463** | -.417** | -.355** | -.421** | -0.088 | .484** | 0.261 | -.471** |
| Peroxynitrite | -0.018 | -0.004 | -.391** | -.514** | -.320* | 0.059 | -0.121 | -.455** | -.476** | -.301* | 0.055 | -.716** | -.643** | -.723** | -.383** | -0.167 | -.686** | 0.162 | .790** | 1 | -.673** | -.732** | -.303* | -0.217 | -0.209 | -.348* | -0.155 | .287* | 0.176 | -.319* |
| Superoxide | 0.085 | 0.013 | 0.201 | .470** | 0.14 | -.312* | .315* | 0.185 | .543** | -0.017 | .313* | .709** | .638** | .643** | .600** | .364** | .666** | -0.182 | -.434** | -.673** | 1 | .422** | -0.081 | -0.167 | -0.092 | 0.187 | 0.215 | -0.002 | 0.017 | 0.005 |
| TBARS | -0.023 | -0.118 | .448** | .379** | .463** | 0.157 | -0.228 | .614** | 0.133 | .623** | -.400** | .452** | .344* | .551** | -0.054 | -0.086 | .426** | -0.258 | -.841** | -.732** | .422** | 1 | .388** | 0.195 | 0.221 | .413** | 0.136 | -.566** | -0.259 | .387** |
| EGC | -0.048 | 0.094 | .276* | 0.157 | .390** | 0.19 | -.340* | .427** | -0.177 | .567** | -.429** | 0.025 | 0.012 | 0.159 | -0.26 | -.339* | 0.004 | -0.158 | -.463** | -.303* | -0.081 | .388** | 1 | .397** | .916** | .274* | -.294* | -.471** | -0.178 | .829** |
| Caffeine | -0.185 | 0.041 | .328* | .292* | .342* | 0.068 | -0.177 | .369** | 0.063 | 0.181 | -0.215 | -0.032 | 0.139 | 0.109 | -0.097 | -0.072 | 0.122 | -0.049 | -.417** | -0.217 | -0.167 | 0.195 | .397** | 1 | .313* | 0.082 | -0.175 | -0.243 | -0.103 | 0.227 |
| C | 0.049 | 0.087 | 0.142 | 0.085 | .306* | 0.097 | -0.268 | .308* | -0.172 | .424** | -.302* | 0.002 | 0.004 | 0.111 | -0.178 | -0.228 | -0.003 | -0.038 | -.355** | -0.209 | -0.092 | 0.221 | .916** | .313* | 1 | .279* | -.404** | -.370** | -0.114 | .850** |
| EC | 0.087 | -0.228 | .285* | .302* | .537** | 0.005 | -.363** | .394** | -0.147 | .493** | -.290* | 0.2 | 0.163 | .320* | -0.167 | -0.152 | 0.144 | -0.139 | -.421** | -.348* | 0.187 | .413** | .274* | 0.082 | .279* | 1 | -.362** | -.419** | -0.144 | .484** |
| EGCG | -0.189 | -0.059 | -0.102 | -0.061 | -0.232 | -0.032 | 0.214 | -0.061 | 0.24 | -0.201 | .371** | .282* | .282* | 0.13 | .376** | .441** | .383** | 0.015 | -0.088 | -0.155 | 0.215 | 0.136 | -.294* | -0.175 | -.404** | -.362** | 1 | .412** | -0.036 | -0.029 |
| ECG | -0.009 | 0.134 | -.439** | -0.161 | -.483** | -.402** | .510** | -.539** | .278* | -.706** | .724** | 0.048 | 0.209 | -0.117 | .588** | .500** | 0.152 | 0.184 | .484** | .287* | -0.002 | -.566** | -.471** | -0.243 | -.370** | -.419** | .412** | 1 | 0.057 | -.297* |
| CG | 0.039 | -0.042 | -0.134 | -0.165 | -0.212 | 0.094 | 0.11 | -0.252 | -0.054 | -.290* | 0.095 | -0.132 | -0.126 | -0.163 | 0.086 | -0.023 | -0.135 | 0.254 | 0.261 | 0.176 | 0.017 | -0.259 | -0.178 | -0.103 | -0.114 | -0.144 | -0.036 | 0.057 | 1 | -0.13 |
| TC | -0.028 | -0.054 | 0.176 | 0.116 | .381** | 0.125 | -.343* | .388** | -0.191 | .504** | -0.25 | 0.126 | 0.116 | 0.201 | -0.133 | -0.114 | 0.146 | -0.085 | -.471** | -.319* | 0.005 | .387** | .829** | 0.227 | .850** | .484** | -0.029 | -.297* | -0.13 | 1 |

*. Correlation is significant at the 0.05 level (2-tailed).

**. Correlation is significant at the 0.01 level (2-tailed)
